# Functionalisation of Polysaccharides for the Purposes of Electrospinning: A Case Study Using HPMC and Si-HPMC

**DOI:** 10.3390/gels1010044

**Published:** 2015-06-30

**Authors:** Jérôme Bodillard, Girish Pattappa, Paul Pilet, Pierre Weiss, Gildas Réthoré

**Affiliations:** 1 LIOAD—Inserm UMRS791—Faculté de chirurgie Dentaire—1, place Alexi Ricordeau 44042 Nantes cedex01, France; E-Mails: jerome.bodillard@gmail.com (J.B.); girish.pattappa@univ-nantes.fr (G.P.); paul.pilet@univ-nantes.fr (P.P.); 2 Centre Hospitalier Universitaire de Nantes, Hotel Dieu, 44042 Nantes, France

**Keywords:** hydrogel, electrospinning, nanofiber

## Abstract

Hydrogels are a suitable scaffold material for a variety of tissue engineering applications. However, these materials have a weak structure and require reinforcement. Integrating electrospun fibers could strengthen material properties. This study created fibers and evaluated the influence of the presence of polar head groups within a polysaccharide backbone following functionalization: silated-hydroxypropyl methylcellulose (Si-HPMC). Electrospinning is a multi-parameter, step by step process that requires optimization of solution and process parameters to understand and control the process. Fibers were created for 2%–3% *wt*/*v* solutions in water and ethanol. Viscosities of solutions were correlated with spinnability. Variations on process parameters did not reveal major variation on fiber morphology. Once controlled, the process was used for HPMC/Si-HPMC mixture solutions. Solubilization and dilution of Si-HPMC were made with common solvents for electrospinning. Two forms of polymer conformation were electrospun: silanol ending and silanolate ending. Microstructures and resulting nanofibers were analyzed by scanning electron microscopy (SEM) and Energy Dispersive Analysis (EDX). The results show the feasibility of our strategy for creating nanofibers and the influence of polar head groups on electrospinnability.

## 1. Introduction

The process of electrospinning has gained rapid interest in recent years due to its potential application in the medical fields of drug delivery and tissue engineering [1]. Electrospinning is a technique used to create polymer nanofibers which can be created from bio-sourced materials, e.g., polysaccharides [2].

The technique was first developed by Formhals and has been subsequently utilized in many tissue engineering applications [3]. Several nanofibers have been manufactured from different polymers with diameter ranging from 40 to 2000 nm [4]. Electrospinning uses electrostatic forces that shape the polymer in solution. Under the influence of an electrostatic field, a drop of the solution is deformed into a cone (Taylor cone) [5] that once the voltage reaches a threshold value, the electrostatic forces overcome the surface tension, a fine charged jet is ejected. The jet is subjected to instabilities (“bending instabilities” [6]) that increase the interface with the environment. This enables drying of the solution and subsequent deposition of polymer as an unwoven fiber mat.

Natural fibers have drawn much attention because they are abundant, biodegradable, renewable, strong, and light weight. Cellulose derivative fibers are valuable for biomedical applications [7,8]. Hydroxypropyl methylcellulose (HPMC) is used as a hydrogel when functionalized with trialcoxy-silane (Si-HPMC) [9]. Applications for cartilage regeneration are under development [10]. Using Si-HPMC electrospun fibers would provide two advantages: insoluble fibers and cross-linkable fibers (via siloxane bonding). Indeed, the objective of the present study was to combine within a single structure, the properties of fibers regarding their form factor properties and the chemical cross-linking properties of the silanol groups of the Si-HPMC hydrogel. Thus, the primary aim is to reinforce and develop new structures through mixing hydrogel and fibers of the same polymer.

This study implements an electrospinning technique using Si-HPMC solutions by studying the influence of salts and ionic head groups within a polysaccharide backbone (HPMC). The ultimate goal is to define methodologies applicable to other functionalized polysaccharides. Initially, electrospinning will be controlled for HPMC according to the literature. Then process and solution parameter variations will be studied and their influence on fiber morphology observed. HPMC and Si-HPMC have different behavior in solution, thus results obtained for HPMC could help in setting up electrospinning parameters for HPMC/Si-HPMC mixtures.

## 2. Results and Discussion

### 2.1. HPMC Solubilization

HPMC powder is an aqueous soluble polymer. Due to its high molecular weight (290,000 g·mol^−1^), solutions are highly viscous at low concentrations. Solutions from 0.1 to 6% *wt*/*v* were prepared. Once dissolved in water, combinations of solvents were formulated (1:1, *wt*:*wt*) in ethanol and THF were obtained. Solutions were prepared at a final concentration of approximately 2.8% *wt*/*v*. To obtain homogeneous solutions, continuous mixing over night was required.

### 2.2. Si-HPMC Solubilization

Si-HPMC powder is soluble in strong basic media (pH 12.4). It is insoluble under magnetic stirring at ambient conditions in THF, DMF, dichloromethane, acetone, and ethanol. Thus, the strategy was to dissolve Si-HPMC in strong basic solution and then dilute the solution with other solvents. The base used by the laboratory is sodium hydroxide (NaOH) at a concentration of 0.2 M. Solutions of Si-HPMC from 3% to 7% *wt*/*v* were created. To extract the excess of tri-silane and methanol created during the dissolution, dialysis was required. Dilutions with the same solvents as previously tested for dissolution were conducted. Amongst these solvents, dichloromethane was not used, as it precipitated the Si-HPMC.

### 2.3. Si-HPMC Solution

**Figure 1:**
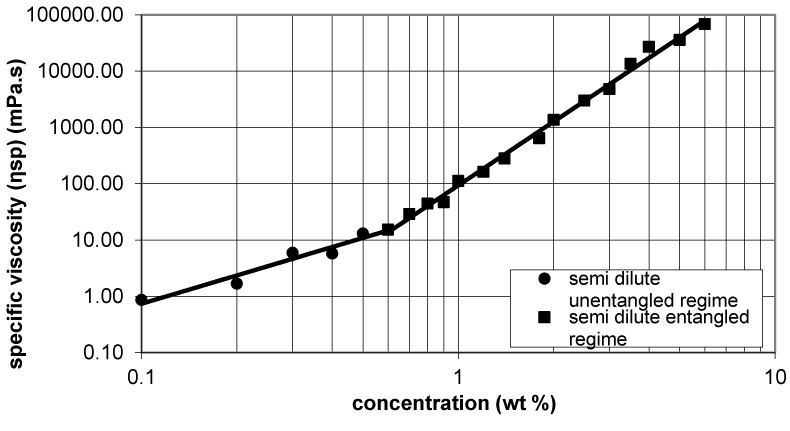
Determination of Ce: specific viscosity as a function of Si-HPMC concentration [11].

Si-HPMC is highly viscous in sodium hydroxide solution. Specific viscosity was used to characterize the outflow of the solution and compared to the literature to determine the range in which the solution could be electrospun. According to McKee *et al.* the range of 2–2.5Ce (Ce is the entanglement concentration) is the minimal range of concentration for electrospinning [11]. Neo *et al.* confirmed this result, obtaining free bead fibers at a concentration of 2.1Ce for zein solution [12]. According to Figure 1, Ce has been determined as 0.6 % *wt*/*v* for Si-HPMC in 0.2 M·NaOH. Ce is the boundary concentration between semi dilute untangled and semi dilute entangled regimes. Indeed, the minimal range has been determined as (1.2; 1.8).

Cross-linking of Si-HPMC solutions is a multi-factorial reaction. The solution-gel transition times vary in accordance with temperature, concentrations, and solvents. To obtain a sufficient quantity of fibers, an electrospinning duration of 1–2 h is necessary. As a consequence, the cross-linking has to be controlled and slower than 2 h. Under the conditions used in the laboratory for injection applications, the gelation time is approximately 40 min. In this case, a HEPES acid buffer is used to promote the neutralization of the solution and then the subsequent cross-linking. It has been observed that a solution with ethanol has a slower gelation (about 3 h), irrespective of an increase in temperature. There should be a competitive reaction between the silanol function grafted on the HPMC and ethanol.

### 2.4. Electrospinning of HPMC

**Figure 2:**
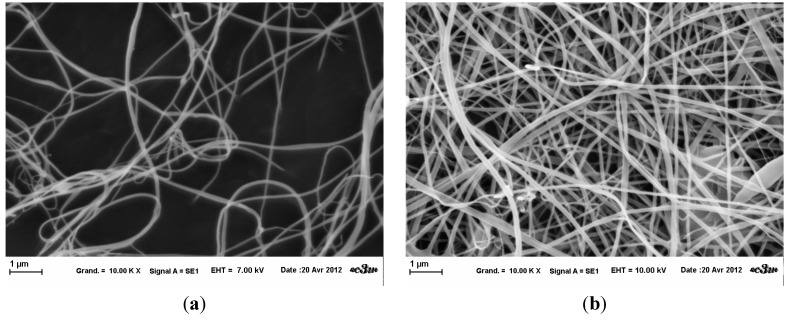

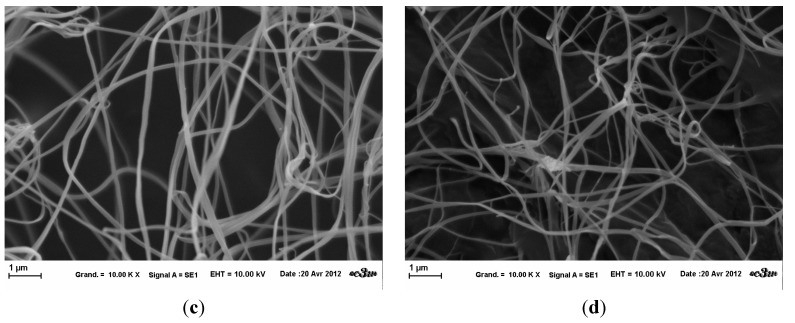
SEM images of HPMC fibers for different concentration at fixed parameters (35 kV, 4.5 mL/H, 20 cm distance and 1 mm tip). (**a**): 2 % *wt*/*v*; (**b**): 2.5 % *wt*/*v*; (**c**): 2.86 % *wt*/*v*: (**d**): 3 % *wt*/*v*.

Initially, fibers were created according to the literature with a flow rate of 4.5mL/h. Variations of the solution were made to understand and control the process. Four solutions with different concentrations were electrospun. Samples were observed at same magnifications at SEM (Figure 2). Differences in the quantity of fibers (Figure 2b) could come from a difference of wrenching. Indeed, it is the only sample for which a continuous piece of mat was taken. Without any statistical measurement, fibers obtained from solution with 2% *wt*/*v* (Figure 2a) were thinner than others. The 2.5 % *wt*/*v* solution fibers (Figure 2b) had a well circular diameter, whereas those at higher concentration were more ribbon-like (Figure 2c,d). These observations must be put into perspective because there were some variations in sample, due to poor evaporation of solvent resulting from drop projection (not shown here). The mean diameter of fibers from 2.86 % *wt*/*v* concentration was measured to be approximately 160 nm (*n* = 40).

### 2.5. Analogy Between HPMC and Si-HPMC

**Figure 3:**
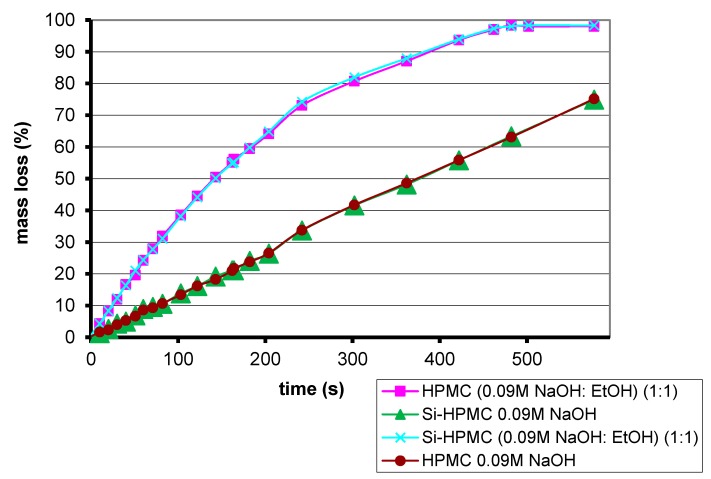
Percentage mass loss through evaporation for 2% *wt*/*v* polymer solutions.

Electrospinning has never been applied to Si-HPMC, an analogy with HPMC was required to set up initial conditions. Comparisons between Si-HPMC and HPMC enable an understanding of which parameters have change to manage fiber preparation. The evaporation of solvent is similar for both solutions of polymers (Figure 3). Four samples were incubated at 37 °C. Ethanol evaporates faster than sodium hydroxide solution. No differences were observed between Si-HPMC and HPMC solutions, which mean that silanization does not influence or prevent solvent evaporation.

The influence of non-modifiable parameters was investigated on HPMC solutions in order to study the electrospinning technique through a step by step process. The influence of sodium hydroxide has been studied on HPMC solutions. HPMC dissolved in 0.09 M·NaOH: ethanol mixture (1:1; 1:0.66; *wt*:*wt*) has been successfully electrospun at different concentrations and for different processing parameters. The ions (Na^+^ and OH^−^) had no critical influence.

**Figure 4:**
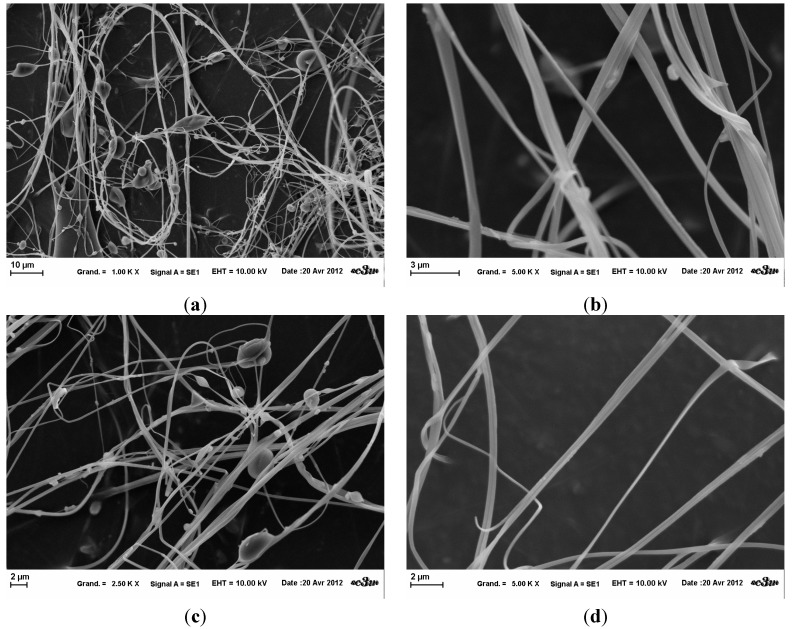
Influence of HEPES buffer on HPMC electrospinning at fixed parameters (35 kV, 4.5 mL/H, 20 cm distance and 1 mm tip). (**a**) at magnification 2.5 k×; (**b**) at magnification 5 k×; (**c**) at magnification 2,5 k×; (**d**) at magnification 5 k×.

To prepare sol-gel electrospinning, the influence of the HEPES based buffer was studied. A 4.3% *wt*/*v* HPMC (0.09 M NaOH: EtOH) (1:1) (*wt*:*wt*) was neutralized with HEPES buffer (volume bending 1v0.5) and electrospun at varying process parameters (Table 1). In both attempts, the same fiber orientation described by Frenot *et al.* was observed [13]. The fibers fixed on the collector only by one extremity after projection, stood straight up in the electrical field. SEM images revealed the creation of micron fibers (Figure 4). However, a lot of salt inclusions and beads were present within the fiber network. There are beaded fibers and ribbon-like fibers, which show the variability of the fibers obtained in these conditions.

**Table 1:** Electrospinning parameters to study HEPES buffer influence.

HPMC [c] % (*wt*/*wt*)	Solution and Solvent	Tension (kV)	Flow Rate (mL/h)	Capillary Tip (mm)	Gap Distance (cm)	Duration (mn)	Observations Figure 4
2.9	4.3 % *wt*/v HPMC (0.09M NaOH: EtOH) (1:1) (*wt*:*wt*) neutralized 1v0.25. Final pH 6.5.	35	4.5	1	20	5	Photo a and b
2.9		30	3.0	0.8	14	30	Photo c and d

### 2.6. Electrospinning of HPMC/Si-HPMC Mixtures

Si-HPMC electrospinning parameters were based on those studied previously. In brief, injection flow rate was at 3.8 mL/h, needle diameter 0.6 mm, 15 cm^2^ aluminum foil acted as a collector and a gap distance of 15 cm.

The mixture of HPMC (Methocel™ E4M) and Si-HPMC will be subsequently referred to as “Template Solutions”. Three different template solutions were successfully electrospun, each containing different proportions of each component (Table 2). The presence of nanofibers containing Si-HPMC was verified via Scanning Electron Microscopy (SEM) observation and Energy Dispersive Analysis (EDX).

It can be observed that a continuous jet projection cannot take place below a certain voltage applied to the generator. If the applied voltage is lower than this limit, there was only projections of droplets (electrospraying). In fact, the jet undergoes a capillary breakdown during the bending instabilities due to its surface tension. If the applied voltage is increased, there will be continuous projections with few droplet projections. During electrospinning, formation of vertical fibers up to a few centimeters long was observed on the collector screen. These fibers collapsed back to the collector, once the high tension is switched off.

**Figure 5:**
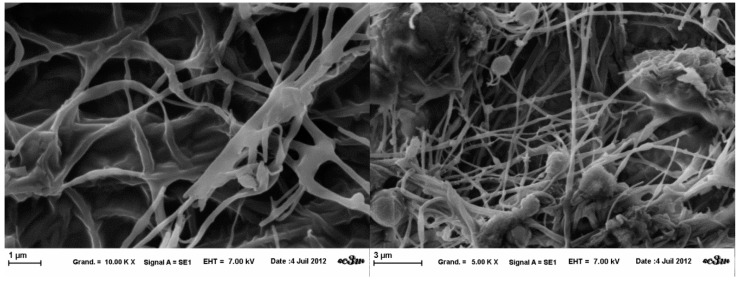
SEM images of template A nanofibers obtained at fixed parameters (35 kV, 4.5 mL/H, 20 cm distance and 1mm tip), at 5 k× magnification (**left**) and 10 k× (**right**).

**Figure 6:**
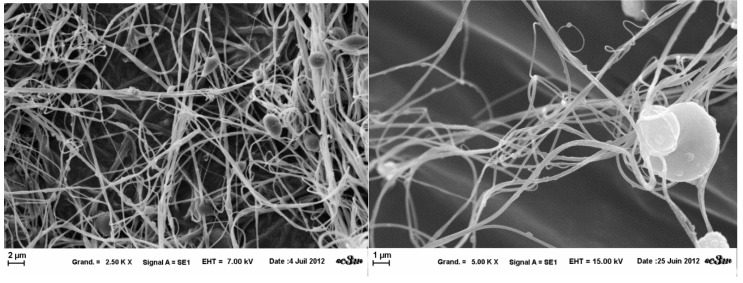
SEM images of template B nanofibers obtained at fixed parameters (35 kV, 4.5 mL/H, 20 cm distance and 1 mm tip), at 2.5 k× magnification (**left**) and 5 k× (**right**).

**Figure 7:**
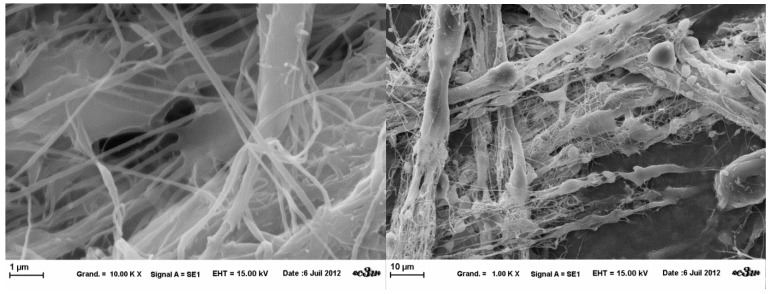
SEM images of template A nanofibers obtained at fixed parameters (35 kV, 4.5 mL/H, 20 cm distance and 1mm tip), at 1 k× magnification (**left**) and 10 k× (**right**).

The fibers obtained from the three template solutions presented a very heterogeneous structure (Figure 5, Figure 6 and Figure 7). Through SEM observations, it was noted that there is coexistence of fibers of different diameters (100 nm–500 nm), and also a beaded-structure.

### 2.7. EDX Analyzes

**Figure 8:**
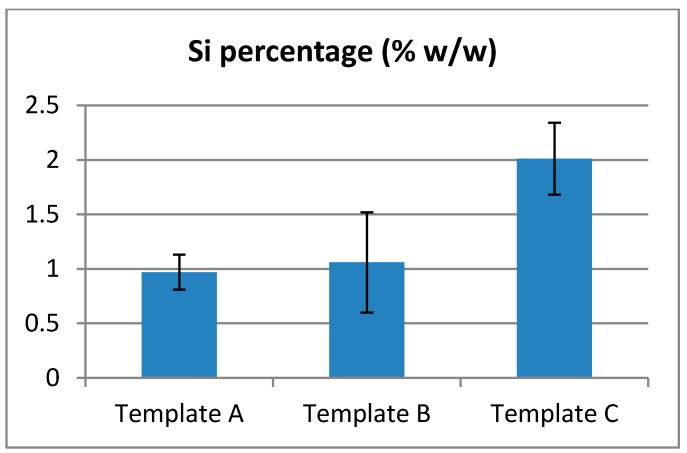
Rate of Silicon detected on electrospun fibers.

EDX Analyzes showed the presence of Silicon amongst the fibers collected (Figure 8). However, due to the large discrepancies in the data, no conclusive quantitative results can be made about the quantity or repartition of Si. It is possible to only conclude that the collected fibers contain Si-HPMC.

According to *Fatimi et al.,* Si-HPMC dissolution strongly depends upon sodium hydroxide concentration [14]. However, it does not change the maximal concentration of polymer that is approximately 5–6 % *wt*/*v*. Only solutions with low concentrations are obtained leading to high viscosity, thus limiting their use for electrospinning. Inhomogeneity was also observed when creating co-solvent solutions during dilution, particularly when the amount of solvent was higher than the 0.09 M NaOH volume. An example was shown when 2.5 % *wt*/*v* Si-HPMC (0.09 M NaOH:THF) (1:1.5) (*wt*:*wt*) formed a solution that included more viscous aggregates.

Electrospinning procedures were reproduced as described in the literature. In terms of HPMC, the influence of process parameters were not as important as previously described [15]. It has been found that a range of parameters enables fiber fabrication with little influence on morphology. Beaded fibers, ribbon-like fibers and small fiber bridges were observed at the same time. However, a limiting parameter for HPMC was the concentration. If the quantity of polymer was low (less than 2 % *wt*/*v* for HPMC), fibers were not created because of lack of interactions between the macromolecules. On the contrary, if the quantity of polymer was too high (more than 3.5 % *wt*/*v*), the viscosity was too high, preventing injectability and thus preventing the fiber formation.

Macroscopic mat appearance seems to differ in homogeneity. If drops did not evaporate, their projection on the collector partially dissolves the mat. Thus, the evaporation of the solvent controlled the appearance of the mat. Despite optimal conditions, in certain instances, the macroscopic appearance of the mat was not controlled. This could be due to drying of the solution at the needle tip. As the Taylor’s cone elongates and solution builds up at the needle tip, drop formations are promoted. The size of the aluminum foil used as a collector appears to help the formation of a steady mat. Consequently, more polymer needs to be electrospun for retrieval of the mat, this increases the time of the electrospinning experiments.

The main limitations observed were that the polymer concentration and the ability of the solution to dry. The high molecular weight of HPMC and SI-HPMC limits the maximum concentration and consequently the size range of the electrospun fibers. Indeed, only nanometer size fibers could be spun, which fits perfectly within the objectives of the study. However, the ability to dry represents an issue that has to be overcome. Fibers cannot be spun without controlling this parameter, as it depends upon the capability of the polymer to retain water within its backbone and it can be used to modulate specific parameters of the electrospinning device (e.g., tension and gap distance). The hydrophilicity of the polymer and the presence of ionic head groups and salts help to retain water and induce a decrease of the applied tension because of the solution conductivity properties that are increased by the presence of charges. However, despite the lack of homogeneity within beaded fiber mats, due to salt inclusions, no influence is expected on the future application. Indeed, once formulated within a hydrogel, containing up to 98% of water, the salt inclusions are expected to dissolve and only the cross-linked fibers will remain.

A typical organization of HPMC fibers was obtained with the solution containing HEPES buffer. Rather than being projected on the grounded collector as an unwoven mat, fibers stood straight up in the electrical field. In comparison with same observation by Frenot *et al.* for enzymatically treated cellulose, we could conclude that Cl^−^ ions are responsible for this conformation. These anions are attracted by the high positive charge at the tip of the nozzle. Further investigations should be done to study the influence of conformation on fiber morphology.

Our strategy was to use the HPMC E4M as template because its spinnability has been demonstrated and the objectives are to create insoluble and cross-linkable fibers. This study has demonstrated that this strategy is applicable and encourages further insight. Indeed, we have successfully prepared electrospun fibers with template solution regarding the HPMC/Si-HPMC ratio.

Electrospinning is a very complex technique dependent upon many parameters such as the polymer, the polar head groups within the polymer backbone, the concentration, the solvent and co-solvent and the presence of other molecules (salts). Their influence also differ according to the technical parameters (e.g., voltage, temperature, collector-tip distance, humidity, and injection speed). Regarding the data obtained throughout this study, it is worth noting that all of these parameters did not interfere with the electrospinnability on their own but more as one multifactorial parameter. Indeed, solvent, co-solvent, and salts present are important for dryness, fiber quality (e.g., salt inclusions), the polymer structure and more importantly the presence of polar head groups within its structure are key for the entanglement properties at fiber formation.

## 3. Conclusions

Towards our goal to prepare Si-HPMC fibers, we first electrospun HPMC solutions as described in the literature. Different tests enabled us to control process parameters. The influence of processing parameters on the creation of fibers was observed. However, few influences have been noticed on fiber morphology. There were also differences in fibers from the same samples: fibers, beaded fibers, drops. This process also presents lack of repeatability. The influences of solvents was also demonstrated. High conductive solutions with salt (Cl^−^ ions) resulted in straight up fibers rather than unwoven mats. Solutions with more elements, especially with HEPES buffer had many salt inclusions and beads within the network. Based on HPMC experiments, Si-HPMC was successfully electrospun using the native polymer HPMC E4M as a spinning carrier. The determinant problem for Si-HPMC electrospinning may be a solution problem. Further investigations will be carried out on solubilization. Reduced viscosity and increased concentration of polymer in more volatile solvent is a proposed solution. One other method would be warm electrospinning because the behavior of Si-HPMC evolves with temperature. To do these further investigations, improvement of the device will be done to maintain the solution at a desired temperature.

## 4. Materials and Methods

### 4.1. Materials

The hydroxypropyl methylcellulose used in this study is Methocel^®^ E4M Premium from the Dow Chemical Company (Mw = 290,000 g·mol^−1^). As specified by the producer, the methoxyl content is 29% and the hydroxypropyl content is 9.7%, corresponding to an average degree of substitution of 1.9 and molar substitution of 0.23. Tetrahydrofuran (THF), 1,4-Dioxane, absolute ethanol acetone, Chloroform, Dimethylformamide (DMF) were purchased from Sigma-Aldrich (Germany). Sodium hydroxide (NaOH) and monosodium phosphate (NaH_2_PO_4_) were purchased at VWR international (France).

### 4.2. Si-HPMC Synthesis

Silane grafting on HPMC involves a Williamson reaction between the hydroxyl function of HPMC and the epoxide group of silane. Using 420 mL of 1-propanol (Acros, Belgium), 1.9l of n-heptane (Acros, Belgium) were stirred. While stirring, 12 g NaOH and 240 g dry HPMC were added to the mixture. The mixture was kept at room temperature for 50 min under nitrogen bubbling. 36 µL of 3-glycidoxypropyltrimethoxysilane (GPTMS) (Aldrich, Germany) (group to be grafted) were added dropwise and temperature was increased to 85 °C. The solution was kept boiling for 3.5 h. Heating was closed and at 40 °C, 30 mL glacial acetic acid was poured to neutralize the reaction. Following 30 min, the mixture was filtered on a buschner. The powder was washed successively four times with 3 L of an acetone/water mixture (85:15 *v*/*v*) to eliminate unreacted GPTMS and HPMC-Si powder was dried at 37 °C. The silane percentage commonly used is 0.59 % *wt*/*v* [16].The principle and procedure of the synthesis of Si-HPMC has been described in detail by Bourges *et al.* [9].

### 4.3. Si-HPMC Solubilization and Solutions

Si-HPMC powders are commonly soluble at basic pH, e.g., sodium hydroxide (NaOH), dilute in solution at a concentration of 0.2 M. Different methods to dissolve Si-HPMC in various solvents were tested (THF, 1,4-Dioxane, ethanol absolute, acetone, Chloroform, DMF). Dissolutions were made under magnetic stirring or ultrasound treatment at room temperature. Si-HPMC powder at 3%, 4%, 5% and 6 % *wt*/*v* was dissolved in 0.2 M NaOH solution (pH = 13). The resulting Si-HPMC solution was dialyzed twice against NaOH solution (0.09 M) using a 6–8 kDa D-Tube Dialyzer (Spectra/Por^®^, UK). The pH value after dialysis was 12.4. These Si-HPMC solutions were then diluted with the above described solvents. Injectability and homogeneity of solutions were studied to prepare electrospinning attempts. Si-HPMC gels were made through mixing with a buffer. The most common is a HEPES buffer developed in our laboratory [9]. This solution is made by dissolving 6.2 g HEPES, 1.8 g sodium chloride (NaCl), in 60 mL 0.1 M·HCl and then diluted in distilled water to reach a final volume of 100 mL. The final buffer pH is approximately 3.5 and blending the solutions initiates cross-linking (Si-HPMC: HEPES buffer) (1:0.5) (*v*:*v*).

### 4.4. HPMC E4M/Si-HPMC Mixtures Preparation

Colorcon Ltd’s Methocel™ E4M was employed as received. Si-HPMC was prepared according to a previously described protocol. The template solutions are detailed in Table 2. Each of these template solutions was dissolved in 0.2 M NaOH and then mixed with pure ethanol, for electrospinability purposes, (weight proportion 1:1) to obtain a final solution of 3% weight. These solutions were placed within a 10 mL PTFE syringe made (BD) for electrospinning. All air-bubbles introduced during the processes were eliminated by ultrasound treatment.

**Table 2:** Polymer proportions of templates solutions.

	Polymer Composition
Template A	1/3 P240KG + 2/3 E4M
Template B	1/2 P240KG + 1/2 E4M
Template C	2/3 P240KG + 1/3 E4M

### 4.5. Electrospinning Device

The electrospinning system used for the experiments is the electrospinning starter kit (reference GHP 5711 63b Linari Biomedical, Italia). The high voltage generator is a HVG-P60-R-EU and the syringe pump, the Razel R99-E, with a 10 mL plastic syringe used (BD Syringe). Metal blunt end needle with 3 diameters were tested: 22G; 0.8 mm and 1 mm. PTFE tubing (Sigma Aldrich) bounded the syringe and the needle. Connections between tubing, syringe and needle were made with male and female Luer lock to tube adapter (Perfektum^®^ needle-tubing connector, Sigma Aldrich). The solution was placed into the spinneret and a high voltage was applied to the solution. The applied voltage was set between 10 kV and 40 kV. The distance between the spinneret and the fiber collector was controlled from 7 cm to 25 cm. The flow rate of the polymer varied from 0.5 mL/h–10 mL/h. The collector was covered by a thin aluminum foil (15 × 15 cm^2^) to enhance conductivity. The experiments were performed at ambient temperature and humidity. Different solutions of HPMC and Si-HPMC were electrospun. The polymer concentration was between 2% and 4 % *wt*/*v*. Upon completion of the spinning process, the non-woven mat was removed from the collector and either characterized by scanning electron microscopy (SEM).

### 4.6. Rheological Measurements

Rheological measurements were performed using a Rheo stress 300 rheometer (ThermoHaakes^®^, Germany) equipped with titanium cone-plate geometry (60 mm diameter, 1° cone, 52 μm gap). The rheometer was equipped with a circulating water bath to control the temperature. The flow curves (viscosity against shear rate) were established for Si-HPMC and HPMC solutions at different concentrations. The experiment was carried out during 180–300 s with 300–500 data points linearly captured in the rate-controlled mode from 0.01 s^−1^ to 8000 s^−1^ with a logarithmic distribution, at room temperature. The flow curves were fitted using the simplified Cross equation:

(1) $$\text{η}=\frac{{\text{η}}_{0}}{1+{\left(\text{λ}\dot{\text{γ}}\right)}^{n}}\;$$

where η_0_ is the limiting Newtonian viscosity at low shear; λ is the relaxation time (the inverse of a critical shear rate γ_c_); n is the exponent of the power law and η the apparent viscosity in Pa·s. Different flow curves were fitted and extrapolated to lower shear rates by the Cross equation to determine the limiting Newtonian viscosity. This value allows characterization the solution by calculation of the specific viscosity (η_sp_) where η_solvent_ is the viscosity of the solvent used.

(2) $${\text{η}}_{sp}=\frac{\text{η}-\;{\text{η}}_{solvent}}{{\text{η}}_{solvent}}\;$$

### 4.7. Optical Microscopy

The microscopic organization of the fiber mats was studied by optical microscopy (Axioplan 2, Zeiss, Germany). Images were taken with DX 40 camera (Kappa, Germany) and imported with Kappa imageBase software (Kappa, Germany). Images at magnifications of ×50, ×100, ×200 and when possible ×400 were taken. The microscope was used in transmission mode when the mat of fibers was removed from aluminum foil and placed between the glass slides. Otherwise, fibers were left on the aluminum foil and analyzed by reflection. When the aluminum foil was used, polymer deposition and the aluminium surface were both observed.

### 4.8. Scanning Electron Microscopy

Polymer fiber samples were taken from mats collected on aluminum foil due to double-face sticky-discs (diameter 1 mm). Samples have been put on metal discs to promote metallization by gold-palladium sputtering (Desk III, Denton Vacuum, United-States). That was conducted under vacuum for 2 min. SEM analyses were carried out using a LEO 1450 VP (Zeiss, Germany). The SEM images were obtained using secondary electron (SE) and if necessary backscattered electron (BSE) modes. SE mode provides information about morphology and surface topography. BSE mode provides information about morphology. The grey level of the image depends upon the atomic weight and concentration of the constituents. It also enables us to localize different chemical species. SEM operating conditions were 10 kV or 15 kV accelerating voltage and optimized working distance for sharpness. Images were taken from magnification ×100–×15000 depending upon the sample studied. Generally, to compare samples, images were made at three magnifications: ×2500, ×5000 and ×10000.
